# Job Satisfaction and Chronic Stress of General Practitioners and Their Teams: Baseline Data of a Cluster-Randomised Trial (IMPROVE*job*)

**DOI:** 10.3390/ijerph18189458

**Published:** 2021-09-08

**Authors:** Lukas Degen, Karen Linden, Tanja Seifried-Dübon, Brigitte Werners, Matthias Grot, Esther Rind, Claudia Pieper, Anna-Lisa Eilerts, Verena Schroeder, Stefanie Kasten, Manuela Schmidt, Julian Goebel, Monika A. Rieger, Birgitta M. Weltermann, on behalf of the IMPROVEjob Consortium

**Affiliations:** 1 Institute of General Practice and Family Medicine, Medical Faculty of the University of Bonn, Venusberg-Campus 1, 53127 Bonn, Germany; karen.linden@ukbonn.de (K.L.); stefanie.kasten@ukbonn.de (S.K.); manuela.schmidt@ukbonn.de (M.S.); julian.goebel@ukbonn.de (J.G.); birgitta.weltermann@ukbonn.de (B.M.W.); 2 Department of Psychosomatic Medicine and Psychotherapy, University Hospital Tuebingen, Osianderstraße 5, 72076 Tuebingen, Germany; tanja.seifried@med.uni-tuebingen.de; 3 Institute of Management, Operations Research, Ruhr University Bochum, Universitätsstr. 150, 44801 Bochum, Germany; or@rub.de (B.W.); matthias.grot@rub.de (M.G.); 4 Institute of Occupational and Social Medicine and Health Services Research, University Hospital Tuebingen, Wilhelmstr. 27, 72074 Tuebingen, Germany; esther.rind@med.uni-tuebingen.de (E.R.); monika.rieger@med.uni-tuebingen.de (M.A.R.); 5 Institute for Medical Informatics, Biometry and Epidemiology, University Hospital of Essen, Hufelandstr. 55, 45147 Essen, Germany; claudia.pieper@uk-essen.de (C.P.); anna-lisa.eilerts@uk-essen.de (A.-L.E.); 6 Center for Clinical Trials, University Hospital Essen, University of Duisburg-Essen, Hufelandstr. 55, 45147 Essen, Germany; verena.schroeder@uk-essen.de

**Keywords:** job satisfaction, perceived psychological stress, primary care, general practices, participatory intervention, psychological wellbeing, leadership, structural prevention, behavioural prevention

## Abstract

*Background*: A high prevalence of poor job satisfaction and high chronic stress is documented for general practitioners (GPs) and non-physician practice staff from various countries. The reasons are multifactorial and include deficits in leadership, communication and workflows. This publicly funded study evaluates the effectiveness of the newly developed participatory, interdisciplinary, and multimodal IMPROVE*job* intervention on improving job satisfaction among GPs and practice personnel. Here, we report the baseline characteristics of the participating GPs and practice assistants, focusing on job satisfaction and perceived chronic stress. *Methods*: The IMPROVE*job* study was performed as a cluster-randomised, controlled trial (cRCT) with German GP practices in the North Rhine Region. The IMPROVE*job* intervention comprised two leadership workshops (one for practice leaders only; a second for leaders and practice assistants), a toolbox with supplemental printed and online material, and a nine-month implementation phase supported by IMPROVE*job* facilitators. The intervention addressed issues of leadership, communication, and work processes. During study nurse visits, participants completed questionnaires at baseline and after nine months follow up. The primary outcome was the change in job satisfaction as measured by the respective scale of the validated German version of the Copenhagen Psychosocial Questionnaire (German COPSOQ, version 2018). Perceived chronic stress was measured using the Trier Inventory of Chronic Stress (TICS- SSCS). *Results*: Recruitment of 60 practices was successful: 21 were solo, 39 were group practices. At baseline, *n* = 84 practice owners, *n* = 28 employed physicians and *n* = 254 practice assistants were included. The mean age of all participants was 44.4 (SD = 12.8). At baseline, the job satisfaction score in the total sample was 74.19 of 100 (±14.45) and the perceived chronic stress score was 19.04 of 48 (±8.78). Practice assistants had a significantly lower job satisfaction than practice owners (*p* < 0.05) and employed physicians (*p* < 0.05). In the regression analysis, perceived chronic stress was negatively associated with job satisfaction (b= −0.606, SE b = 0.082, *p* < 0.001, ICC = 0.10). *Discussion*: The degree of job satisfaction was similar to those in other medical professionals published in studies, while perceived chronic stress was markedly higher compared to the general German population. These findings confirm the need for interventions to improve psychological wellbeing in GP practice personnel.

## 1. Introduction

Research in several European general practitioner (GP) populations has shown that job satisfaction is linked to work-related factors [1,2,3]. Among the factors known to reduce job satisfaction are excessive work hours, high workload, time pressure, bureaucracy, insufficient salary and lack of appreciation [3]. Ongoing burdening working conditions have been associated with chronic stress, burnout, depression and early retirement. In addition, there is a correlation with poorer patient outcomes [4,5,6,7,8,9,10,11]. Compared to the general population, a study by Viehmann et al. showed that physicians and non-physician staff in German general practices are twice as often affected by self-reported high chronic stress [12].

Multiple reasons for high levels of chronic stress in GPs have been shown, such as insufficient leadership skills, poor work organisation and lack of communication with patients and within the team [13,14]. There are various approaches to reduce chronic stress in a GP setting. For example, a study from Australia showed a reduction of stress levels in a GP sample using a cognitive behavioral coaching program [15]. Fortney et al. showed that a mindfulness intervention reduced stress in primary care physicians [16]. A variety of approaches have been developed and evaluated which aim at improving the mental health of healthcare workers [17]. The majority of the interventions target individual behaviour such as stress management through, e.g., meditation or training of self-care [18,19]. However, based on the European Principles of Occupational Health and Safety, interventions should first target the work environment and focus on individual behavioural prevention thereafter [20]. A review of organisation-related interventions showed that health-promoting effects are enhanced if the interventions simultaneously focus on working conditions, work processes and work equipment [21,22]. Based on such evidence, we designed the IMPROVE*job* intervention to improve job satisfaction among German general practice personnel focusing on the aspects of leadership, communication, and work processes with an organisational change approach.

The IMPROVE*job* intervention comprised two workshops, one for GPs with leadership responsibilities and one for GPs and other practice personnel, educational material as well as an implementation phase of nine months supported by IMPROVE*job* facilitators. The study was initiated by an academic general practitioner. The intervention itself was developed using a participatory approach: experts from the fields of general practice and family medicine cooperated with those from operations research, occupational and psychosomatic medicine, health promotion and epidemiology to develop a target-group-oriented multimodal intervention; general practitioners and practice assistants were regularly asked to provide input and feedback in order to tailor the intervention to the needs of general practices. The intervention addressed central potential determinants of job satisfaction and chronic stress in GP practices, namely leadership, communication, work organisation and workflows, workplace health promotion and occupational health and safety [22]. The IMPROVE***job*** intervention is of relevance as the problem of chronic stress in the primary care workforce has not been solved.

The effectiveness of the intervention on job satisfaction is studied in a cluster-randomised trial with personnel from German practices. Details on the study protocol have been published [22]. Here, we report the baseline characteristics of the participating practices, GPs and practice assistants, focusing on job satisfaction and perceived chronic stress.

## 2. Materials and Methods

### 2.1. Study Design

**Figure 1:**
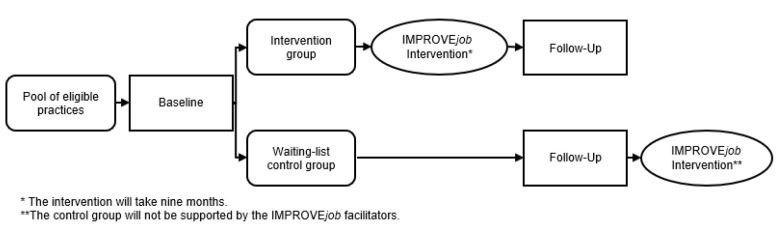
Study design [22].

The IMPROVE*job* study was performed as a cluster-randomised, controlled trial (cRCT) with personnel from GP practices who were randomised in an intervention and a control group (Registration number: DRKS00012677). The control group was conducted as a waiting list control group, i.e., these participants received the intervention after the collection of follow-up data. The study aims at evaluating the effectiveness of the IMPROVE*job* intervention on increasing job satisfaction as measured by the German version of the COPSOQ 2018 (primary outcome). Study nurses visited practices for the collection of baseline data prior to randomisation. The intervention lasted nine months. For details, see Figure 1.

### 2.2. Target Population: Practices and Practice Personnel

The study population consists of practice personnel of general practices of the North Rhine Region in Germany. The details are outlined in our study protocol [20]. As background information on the German healthcare system, GPs typically are the first point of contact for patients in primary care and provide an interface with the rest of the healthcare system, yet initial contacts to secondary care is also possible for patients. GP practices are small businesses most frequently owned by primary care physicians, who may employ other physicians and practice assistants [23].

The following inclusion and exclusion criteria were applied. Practices were included (1) If their owner was registered as general practitioner of the Association of Statutory Health Insurance Physicians of North Rhine with or without affiliation as teaching practice of the University of Bonn or the University of Cologne. (2) If the practice owner and at least one practice assistant provided informed consent for study participation. We aimed at recruiting all members of a participating practice team including physicians and practice assistants in training. Practices were excluded if special situations such as a relocation of the practice or retirement of the owner were imminent. Additionally, practices that had participated in the development of the IMPROVE*job* intervention or participated in the feasibility study of the intervention were excluded.

According to the sample size calculation (for details, see [22]), a total of 56 practices with an average of 4 participants per practice were targeted for recruitment, allowing 2 dropouts each in the intervention and control group. The randomisation took place after baseline data collection and was performed by an independent researcher of the Centre for Clinical Studies Essen. It was stratified for (a) single/group practice and (b) teaching/non-teaching practice.

### 2.3. Recruitment and Non-Responder Analysis

**Figure 2:**
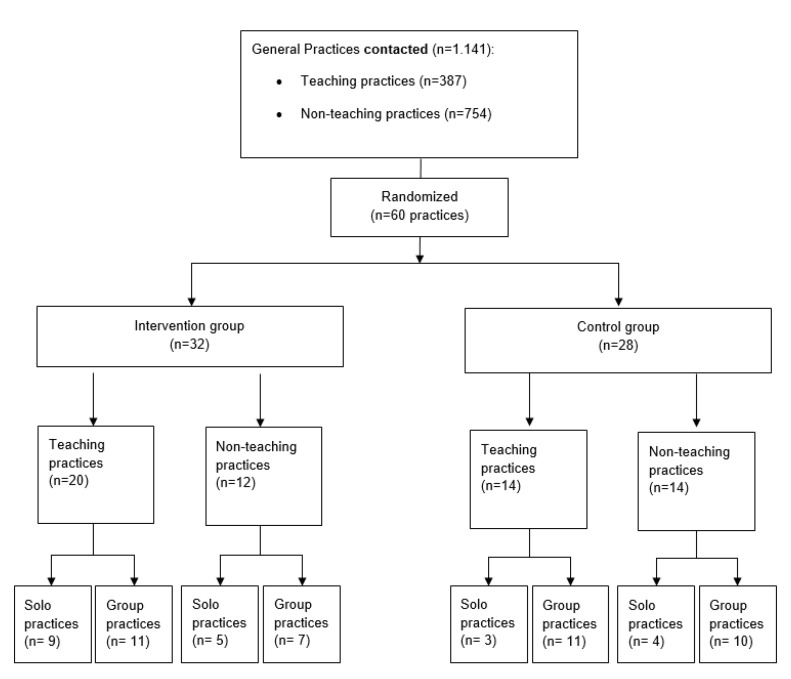
Recruitment flow chart.

Practice recruitment was carried out by the Institute of General Practice and Family Medicine of the University of Bonn. Invitations were sent by letter, fax or e-mail including participant information and the practice consent form to be signed by the practice owner. An incentive of EUR 50 was offered per participant completing the follow-up data collection. If practices did not answer after more than three contacts, reasons for non-responding were gathered by fax. The recruitment process started in September 2019. A total of 1141 practices were contacted—387 teaching and 754 non-teaching practices (for details, see Figure 2)—assuming a higher participation rate among teaching practices. During phone recruitment, practices frequently voiced interest in the topic but felt unable to participate in three workshops within six weeks as originally planned (for details, see [22]). The practice assistants in particular considered two team workshops as too time consuming. Of the 1141 practices contacted, a total of 60 practices agreed to participate. Detailed written non-responder information was available for 288 practices and showed that ‘no interest’ (57%), ‘no time’ (23%) and ‘no need’ (9%) were the most common reasons for non-participation. Based on the oral feedback during recruitment and the results derived from non-responder analysis, the IMPROVE*job* consortium decided to reduce the intervention from three to two workshop afternoons. This restructuring of the workshops was carried out without loss of content, as some material was newly provided online.

### 2.4. IMPROVE*job* Intervention

**Figure 3:**
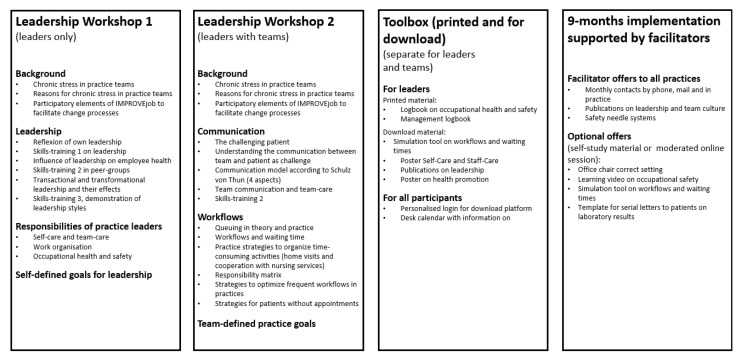
Elements of the IMPROVE*job* intervention.

The final multi-modal IMPROVE*job* intervention consisted of the following elements (Figure 3):

1. Two leadership workshops: one workshop for practice leaders only, and a second workshop for the leaders together with their practice teams;
2. The toolbox with supplemental material: printed and online material including learning videos;
3. The implementation phase of nine months supported by IMPROVE*job* facilitators.

The intervention workshops comprised four hours each on Wednesday afternoons, which were held two weeks apart. For each workshop set, an average of 4 to 6 practice teams was invited. This allowed for about 6 to 10 physicians and 15 to 25 practice assistants per workshop series. A total of 6 workshop sets were carried out between November 2019 and March 2020. The workshops contained presentations by the research team combined with interactive elements, self-reflection, peer exchange, and skills-trainings supported by simulation practice assistants/patients. The latter were recruited from the simulation patient pool of the University Hospital Bonn, and were trained by the researchers. Workshop 1 for physicians with leadership responsibilities (practice owners and employed physicians with leadership responsibilities) addressed the topics ‘role of the executive’, ‘leadership styles’ and ‘occupation health and safety for GP practices’ in theory and practice.

Workshop 2 for physicians with leadership responsibilities and all practice employees focused on the topics ‘work organisation including appointment scheduling’, ‘workplace health promotion’, ‘communication’, and ‘occupational health and safety’.

At the end of workshop 2, each practice decided on self-selected improvement goals. In addition, the toolbox was introduced and an outlook on the upcoming implementation phase supported by IMPROVE*job* facilitators was given.

The toolbox contained content presented in the workshop and additional material: The ‘management logbook’ for physicians with leadership responsibilities, the ‘employee logbook’, the desk calendar for practice teams with multiple contents from the workshops and supplemental material for download.

After workshop 2, the nine-month implementation phase supported by IMPROVE*job* facilitators began. The facilitators were trained members of the research team with professional experience as practice assistants who supported practices in their change processes by giving information via phone/mail and offering up to 5 practice visits with additional material. The main goal of the facilitators was to keep the IMPROVE*job* idea alive for 9 months and to help each practice to achieve their self-defined practice goals.

Due to the COVID-19 pandemic, with a lockdown in Germany starting in mid-March 2020 and the need to abstain from gatherings in larger groups, the last team workshop of the intervention group and all workshops for the control group were performed as online meetings. Therefore, the content was modified for application as interactive online video-sessions; some elements were integrated into an e-learning-platform. Overall, the intervention content remained identical.

### 2.5. Baseline Data Collection and Outcomes

The baseline data collection was performed by trained study nurses from the Center for Clinical Trials, University Hospital Essen, during a prescheduled appointment in the participating practices to assure that all practice personnel were present. Questionnaires differed slightly by professional role (for practice leaders, employed physicians, and practice assistants). In addition, the physician practice owners filled in a questionnaire addressing practice characteristics, such as practice type, number of employees and patients. For details, see [22].

#### 2.5.1. Primary Outcome

The primary outcome of the IMPROVE*job* study was defined as change in job satisfaction, measured by the respective scale of the German version of the Copenhagen Psychosocial Questionnaire based on the international COPSOQ version (German COPSOQ, version 2018). The job satisfaction scale consists of 5 items and is combined with an additional global item (‘How pleased are you with your job as a whole, everything taken into consideration?’) using a 5-point Likert scale. To calculate scores, we followed the recommendation for the COPSOQ transformation: the answering scales were transformed into a score ranging from 0 (minimum value, ‘not satisfied at all’) to 100 points (maximum value, ‘fully satisfied’) [24].

The outcome ’job satisfaction’ was chosen as the primary outcome because the IMPROVE*job* study evaluates a complex intervention that takes into account various factors known to influence the psychological wellbeing.

#### 2.5.2. Secondary Outcomes

Using questionnaires, the following additional aspects were requested from the participants: (1) leadership, (2) general health and work ability, (3) work-related experience and behaviour patterns, (4) perceived chronic stress, (5) occupational safety culture, (6) stress coping strategies applied by participants, (7) work organisational issues including waiting times, team roles and team activities, and (8) team activities and roles. The details are published in our study protocol [20]. This baseline paper describes the following baseline results:

1. Baseline characteristics of the study participants.
2. Job satisfaction as the primary outcome measured with the respective COPSOQ scale.
3. Perceived chronic stress: To measure chronic stress, the German short version of the Screening Scale of the Trier Inventory for the Assessment of Chronic Stress (TICS-SSCS) was used. The TICS-SCSS consists of 12 items using a 5-point Likert scale (range 0–4). All 12 items are added up to a sum (0–48), where 0 is the lowest possible and 48 the highest possible perceived stress in the last 3 months [25].

### 2.6. Statistical Analysis and Ethics’ Statement

A description of the sample was carried out at the individual participant level and at the cluster level (GP practices) in total and stratified by study arms.

Baseline data on the primary outcome (job satisfaction) and perceived chronic stress will be analysed in the whole sample. Subgroup descriptions will be performed in relevant strata, such as (a) practice owner, employed physician and practice assistant and (b) teaching compared to non-teaching practices. All variables will be described using standard descriptive methods appropriate to their measurement level. Parametric measures such as mean and standard deviation are reported to allow for comparability of the results. Subpopulations were compared using T-test statistics. A *p*-value below 0.05 was considered significant.

Following our study protocol [22], the COPSOQ score for job satisfaction (German COPSOQ version 2018) and the TICS-SSCS sum were calculated according to the standards for these scales. In addition, we calculated the respective COPSOQ items of the COPSOQ version 2021, which includes an extra item addressing satisfaction with income. This item was under evaluation when we conducted the study and is now included. A mixed linear regression model was used to calculate the association of perceived chronic stress with job satisfaction, respecting practice clusters. Statistical analyses were conducted with SPSS for Windows, version 25 (IBM Corp., Armonk, NY, USA).

The study was approved first by the Ethics Committee of the Medical Faculty of the University of Bonn (Reference number: 057/19, date of approval: 20/02/2019).

## 3. Results

### Study Populations: Baseline Characteristics of Practices and Practice Personnel

Of all *n* = 60 practices randomised, *n* = 21 were solo practices (*n* = 14 intervention group vs. *n* = 7 control group) and *n* = 39 were group practices (*n* = 18 intervention vs. *n* = 21 control group). *n* = 34 teaching (20 in intervention and 14 in control group) and *n* = 26 non-teaching practices (12 in intervention and 14 in control group) were included.

A total of 366 practice personnel took part at baseline: 84 physician practice owners, 28 employed physicians and 254 practice assistants (Table 1). At the practice level, the percentage of personnel participating ranged from 20.0 to 100% (mean = 73.4%). T-tests showed no statistically significant difference between baseline data of perceived chronical stress (TICS-SSCS), t(359) = 0.778, *p* = 0.437 between the intervention (*n* = 182) and control group (*n* = 179). Additionally, there was no statistically significant difference regarding job satisfaction (German COPSOQ, version 2018) between the intervention (*n* = 180) and control group (*n* = 181), t(361) = −0.463, *p* = 0.644.

At baseline, the mean age of all participants was 44.4 years (±12.8). Practice owners were, on average, ten years older and more likely to work full-time compared to the other two professional groups. The practice owners worked for 15.3 (±8.4) years in the current practice, the employed physicians for 3.9 (±5.4) and the practice assistants for 8.8 (±8.9) years.

As indicated by large standard deviations, practice assistants varied markedly regarding age, training and living situation. On average, *n* = 235 had graduated 19.9 (±13.3) years ago, while *n* = 19 were still in training. Of the practice assistants, 38 were qualified for advanced tasks (so called VERAH/EVA qualification). On average, the practice assistants worked 32.7 (±10.7) hours per week in the last 3 months.

At baseline, the mean job satisfaction score (German COPSOQ, version 2018) in the total sample was 74.19 of 100 (±14.45, Median: 75); using the 7-item scale (COPSOQ 2021), the mean job satisfaction score was 72.58 of 100 (±14.66, Median: 75).

The perceived chronic stress score was 19.0 of 48 (±8.78, Median: 19). Two practice owners were identified as outliers for the job satisfaction score. As they did not significantly skew results, they are included in the following analyses. Practice assistants had a significantly lower job satisfaction than practice owners (*p* < 0.01) and employed physicians (*p* < 0.05). No statistically significant difference was observed between the occupational groups for perceived chronic stress. For details, see Table A1.

**Figure 4:**
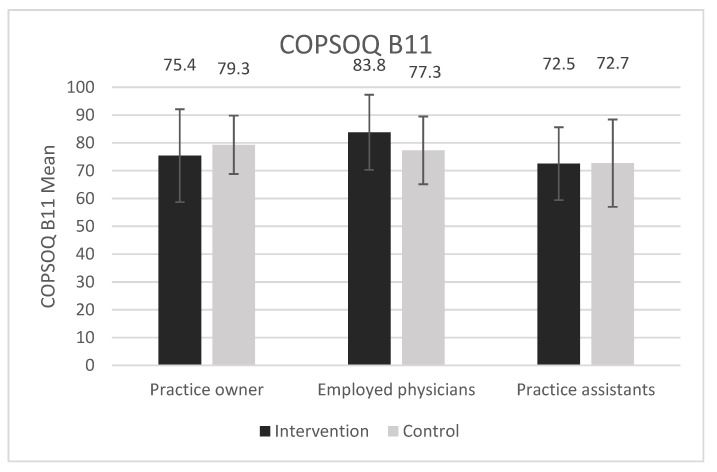
Job satisfaction (German COPSOQ, version 2018): mean scores of practice owners, employed physicians and practice assistants for intervention and control group. Error bars show standard deviations. There are no significant differences between groups at baseline (*p* > 0.05).

**Figure 5:**
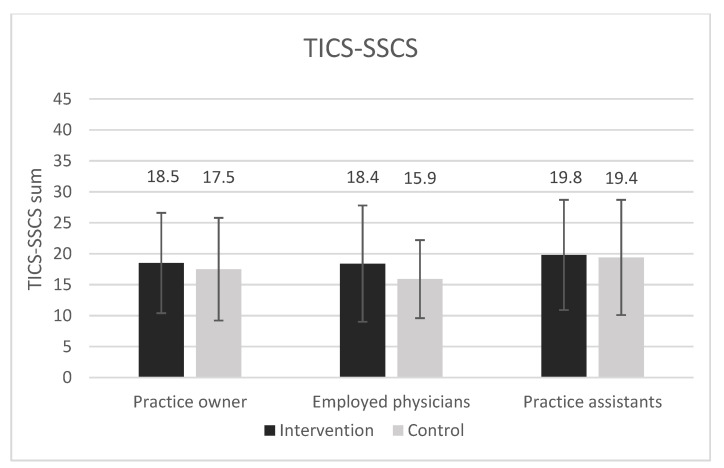
TICS-SSCS sum scores of practice owners, employed physicians and practice assistants are shown for study arms. Error bars show standard deviations. All presented differences between intervention and control group are non-significant (*p* > 0.05).

Figure 4 and Figure 5 show a graphical overview of the main outcome job satisfaction measured with the COPSOQ B11 (German COPSOQ, version 2018) and perceived chronic stress measured with the TICS-SSCS scale stratified by occupational groups and study arms.

Overall, the mixed regression model showed a significant negative association between perceived chronic stress and job satisfaction (German COPSOQ, version 2018) respecting the practice clusters (b = −0.606, SE b = 0.082, *p* < 0.001, ICC = 0.10). There was also no relevant difference when using the COPSOQ 2021 (b = −0.632, SE b = 0.083, *p* < 0.001, ICC = 0.10).

## 4. Discussion

In line with prior studies [12,13,14], we showed a high job satisfaction among GP practice personnel, yet, at the same time, a high level of perceived chronic stress. To our knowledge, this is the first study in this target group that measured both job satisfaction and perceived chronic stress simultaneously. As expected, the results show an inverse relationship between job satisfaction and perceived chronic stress. Comparing physicians and practice assistants, the latter showed higher perceived chronic stress and a significantly lower job satisfaction. Yet, compared to 2017 data of the COPSOQ databank across all occupations, all GP practice personnel showed markedly higher job satisfaction (74.19 vs. 62.3 of 100) [26]. Focusing on the medical field only, practice owners and employed physicians in our study scored higher regarding job satisfaction than more than 2000 hospital physicians documented in the COPSOQ databank (77.16 and 79.61 compared to 62.4). Likewise, practice assistants in our study scored higher than >8000 hospital-based nurses from the COPSOQ databank (72.58 vs. 57.8) [26]. Similar to our results, Goetz et al. demonstrated a high job satisfaction in 523 GPs and 1158 practice assistants using the 10-item Warr–Cook–Wall questionnaire [13,27] rather than the COPSOQ scale, which does not allow for direct comparison. A survey from Denmark showed low job satisfaction for 22.1% of the participating GPs with a limited comparability to our population [28].

A number of studies in GP practice personnel addressed the complex relationship of working conditions and the psychological wellbeing of practice staff. Based on four studies, a literature review indicated that task delegation in general practice is viewed positively by practice staff and contributes to job satisfaction, mainly due to the autonomy perceived [29]. Goetz et al. showed that practice assistants were most satisfied with their team colleagues, but dissatisfied with their income [27]. Practice assistants were more likely to report a higher job satisfaction if a good working atmosphere was present, indicated by, e.g., opportunities to contribute to practice improvement and having defined responsibilities within the practice team. Likewise, Hoffmann et al. identified various factors influencing mental workload, which is defined as the exposure to individual work demands in 550 practice assistants from 130 practices: social support at work and participation were identified as the key protective factors [30]. Our multimodal IMPROVE*job* intervention including workshops and a nine-month implementation phase addressed several of these aspects aiming at reducing the psychological stress and strain for physicians and practice assistants in primary care. Hereby, the burden of perceived chronic stress deserves special attention, as our baseline results show a mean of 19 among all participants as measured by the TICS-SSCS. Stratified by occupational groups, there was a difference between employed physicians (16.79), practice owners (18.06) and practice assistants who had the highest level (19.62). These results are in line with the findings of Viehmann et al. in 2017, which showed high levels of perceived chronic stress, especially among practice assistants within *n* = 136 German GP practices [12]. The perceived chronic stress of GP practice personnel is obvious through a comparison with representative data from the German general population reported in the “German Health Interview and Examination Survey for Adults” (DEGS1): while the DEGS1 showed a median TICS-SSCS score of 11 [31], our target group had a median score of 19 using the same study instrument. Although job satisfaction is relatively high, the high scores for perceived chronic stress demonstrate the potential relevance of the IMPROVE*job* intervention. 

## 5. Strengths and Limitations

After the described reduction in the number of intervention workshops, the target number of practices (*n* = 60) was recruited successfully. Yet, we cannot exclude that practices with very high perceived chronic stress did not participate in the study. The stratified randomisation, with respect to teaching and non-teaching practices as well as solo and group practices, is an important methodological feature influencing the generalisability of the results. Additionally, the baseline characteristics of the practices and participants as well as the scores for job satisfaction and perceived chronic stress did not differ between intervention and control group, which will facilitate the assessment of intervention effects. Subgroup analysis regarding the association of job satisfaction and chronic stress was likely limited due to the small sample of employed physicians. Our sample size was rather large (>360 participants), yet any generalization needs to be handled with caution.

## 6. Conclusions

Compared to data from large German representative samples, GPs and practice assistants in our study showed above average scores for job satisfaction and perceived chronic stress at baseline. Comparison of these baseline results with our follow-up data will show whether these indicators of mental wellbeing in the primary care workforce can be successfully improved by our IMPROVE*job* intervention. In line with other studies, we showed an urgent need to improve mental well-being of the primary care workforce. Any successful approach to reduce chronic stress will have practical implications given the relationship between chronic stress and job satisfaction. Further research to address the complex workplace scenarios in primary care practices is needed.

## Figures and Tables

**Table 1:** Sociodemographic description of participants at baseline.

Sociodemographic Description	Total Sample	Practice Owner	Employed Physician	Practice Assistant
Variable	*n* = 366	*n* = 84	*n* = 28	*n* = 254
Female, %	87.1	52.4	78.6	99.6
Age in years, mean (SD)	44.4 (12.8)	54.3 (6.2)	44.8 (9.8)	41.0 (13.0)
Years in current practice, mean (SD)	10.0 (9.1)	15.3 (8.4)	3.9 (5.4)	8.8 (8.9)
Working full time, %	52.0	90.5	28.6	41.5
Living in a relationship married, %	78.6	87.8	88.9	74.5
Persons in household over 18 years, mean (SD)	2.2 (1.0)	2.1 (1.0)	2.0 (0.5)	2.2 (1.1)
Persons in household under 18 years, mean (SD)	1.2 (1.0)	1.3 (1.3)	1.4 (1.0)	1.0 (0.9)
Care for next-of-kin, %	20.8	21.7	0.0	22.9
Professional characteristics of physicians (*n* = 112)				
Years since accreditation as physician, mean (SD)	24 (9.1)	26.6 (7.2)	16.3 (9.7)	-
Years since licensed for the statutory health insurance, mean (SD)	14.5 (9.4)	16.4 (8.4)	5.8 (8.8)	-
Physician in GP training, %	-	-	25.0	-
Professional characteristics of practice assistants (*n* = 254)				
Years since graduation, mean (SD)	-	-	-	19.9 (13.3)
Qualification as practice assistant, %	-	-	-	81.9
No additional qualification, %	-	-	-	64.2
Practice assistant in training, %	-	-	-	7.5
Average working hours in last 3 months per week, mean (SD)	-	-	-	32.7 (10.7)

## Data Availability

There are no plans to grant access to full protocol, participant-level dataset or statistical code as data contain potentially identifying information.

## Appendix A

**Table A1:** Job satisfaction (German COPSOQ, version 2018) and perceived chronic stress: total sample and stratified by profession as well as study arm.

Job Satisfaction and Perceived Chronic Stress, Stratified	Total Sample								Intervention Group						Control Group					
	N	Total	N	Practice Owner	N	Employed Physician	N	Practice Assistant	N	Practice Owner	N	Employed Physician	N	Practice Assistant	N	Practice Owner	N	Employed Physician	N	Practice Assistant
Job Satisfaction, mean (SD)	363	74.19 (14.45)	84	77.16 (14.30)	28	79.61 (12.85)	251	72.58 (14.42)	46	75.37 (16.69)	10	83.75 (13.53)	126	72.49 (13.14)	38	79.33 (10.55)	18	77.31 (12.23)	125	72.68 (15.65)
TICS, mean (SD)	361	19.04 (8.78)	83	18.06 (8.15)	28	16.79 (7.45)	250	19.62 (9.07)	46	18.54 (8.10)	10	18.40 (9.35)	126	19.79 (8.92)	37	17.46 (8.29)	18	15.89 (6.28)	124	19.44 (9.26)
